# In Times of Stress, Mutate Early and Often

**DOI:** 10.1371/journal.pbio.0020438

**Published:** 2004-11-23

For a human, the normal response to stress is to reduce it through some purposeful action, be it indulging in chocolate or calling in sick, at a rate which we can vary to fit the circumstances. For a strain of bacteria faced with stress, the choice is often more stark: it must mutate or die. Among evolutionary theorists, an important question has been whether the rate of mutation is fixed, or instead can adaptively increase in response to stress, thereby increasing the likelihood of a favorable mutation. Something like this latter possibility was envisioned by Darwin, but fell out of favor among some neo-Darwinists, for whom a steady rate of mutation was more in keeping with their overall model of evolutionary gradualism. This debate is taken up in a new study in this issue by P. J. Hastings and colleagues, who examined the mechanism by which Escherichia coli lacking the ability to digest lactose, called *lac ^−^* mutants, regain that ability when presented with lactose as their only food source.

It has been known for some time that the reversion of *lac ^−^* mutants to a *lac^+^* state can be achieved by either of two genetic events: amplification, which creates numerous copies of the nonfunctional *lac* gene, and point mutations, which give rise to functional versions of the gene (many non-useful mutations also occur; thus, there is no directed mutation, in keeping with standard Darwinian evolution). According to the gradualist view, amplification precedes mutation, and the rapid appearance of *lac^+^* cells is explained by a normal mutation rate acting on multiple copies of the gene. In contrast, according to the hypermutation view, amplification and mutation are independent events, and *lac^+^* cells arise quickly because the mutation rate has increased.

While some results from previous studies have supported the gradualist interpretation, the experiments of Hastings et al. show that hypermutation is the most plausible explanation. A variety of procedural improvements allowed them to analyze more individual cells at an earlier stage of colony development. For instance, they analyzed colonies composed of as few as one hundred cells, rather than the ten thousand cells in prior experiments, and even nascent colonies at the two-cell stage.[Fig pbio-0020438-g001]

**Figure pbio-0020438-g001:**
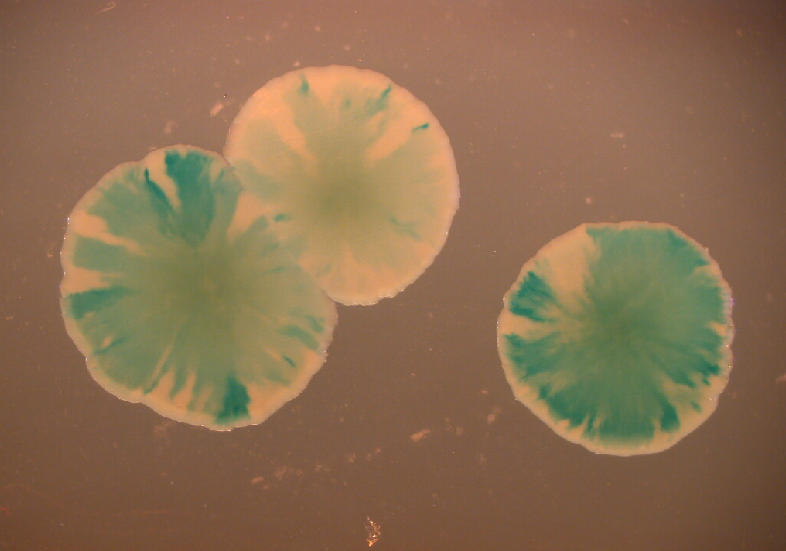
Sectored colonies of *lac ^+^* and *lac^−^* E. coli

The study produced clear evidence that point mutations arise very early in the development of *lac^+^* colonies, before amplification can account for the number of *lac^+^* revertants observed. Amplification is not only independent from mutation, but occurs relatively late under starvation. The researchers found that amplification, but not point mutation, requires the presence of a particular DNA polymerase, further strengthening the case that amplification need not precede mutation. They also showed that amplification by itself does not induce a so-called SOS response. The SOS system includes a group of genes that cause an increase in mutation in response to stress, and one hypothesis arising from the gradualist model was that amplification turned on the SOS response.

Based on their data, the authors reject the strict gradualist model for the adaptive mutation mechanism in the Lac system. They propose that amplification and hypermutation are independent responses to stress, each of which increases the likelihood of adaptive change. They also suggest that a stress-induced increase in the rate of point mutations may have implications for a variety of mutation-related phenomena, from tumor formation to development of resistance to antibiotics and chemotherapeutic drugs.

